# Shiga Toxins: Intracellular Trafficking to the ER Leading to Activation of Host Cell Stress Responses

**DOI:** 10.3390/toxins2061515

**Published:** 2010-06-17

**Authors:** Moo-Seung Lee, Rama P. Cherla, Vernon L. Tesh

**Affiliations:** Department of Microbial and Molecular Pathogenesis, Texas A & M Health Science Center, 407 Joe H. Reynolds Medical Building, College Station, TX, 77843-1114, USA; Email: mlee@medicine.tamhsc.edu (M.-S.L.); rpcherla@medicine.tamhsc.edu (R.P.C)

**Keywords:** Shiga toxins, retrograde transport, ribotoxic stress response, ER stress response, apoptosis

## Abstract

Despite efforts to improve hygenic conditions and regulate food and drinking water safety, the enteric pathogens, Shiga toxin-producing *Escherichia coli* (STEC) and *Shigella dysenteriae* serotype 1 remain major public health concerns due to widespread outbreaks and the severity of extra-intestinal diseases they cause, including acute renal failure and central nervous system complications. Shiga toxins are the key virulence factors expressed by these pathogens mediating extra-intestinal disease. Delivery of the toxins to the endoplasmic reticulum (ER) results in host cell protein synthesis inhibition, activation of the ribotoxic stress response, the ER stress response, and in some cases, the induction of apoptosis. Intrinsic and/or extrinsic apoptosis inducing pathways are involved in executing cell death following intoxication. In this review we provide an overview of the current understanding Shiga toxin intracellular trafficking, host cellular responses to the toxin and ER stress-induced apoptosis with an emphasis on recent findings.

## 1. Introduction

Shiga toxins (alternatively called verotoxins or Shiga-like toxins) are major virulence factors expressed by the enteric pathogens *Shigella dysenteriae* serotype 1 and certain serotypes of *Escherichia coli*. Shiga toxin-producing bacteria constitute a significant public health concern in developing and developed countries. In particular, the presence of Shiga toxin-producing *Escherichia coli* (STEC) in the food supply may cause widespread outbreaks of disease. Infection of humans with as few as 10–100 organisms of *Shigella dysenteriae* serotype 1 or STEC may initially cause bloody diarrhea which may progress, particularly in children and the elderly, to a form of acute renal failure which is called the hemolytic uremic syndrome (HUS), and may be followed by neurological complications in severe cases. HUS is characterized by the clinical triad of thrombotic microangiopathy, hemolytic anemia and thrombocytopenia [1,2,3]. Nervous system complications may include lethargy, disorientation, seizures, and paralysis [4]. The toxins appear capable of crossing the intact intestinal epithelial barrier via transcytotic or paracellular mechanisms [5,6], damage the colonic microvasculature [7,8], may then associate with blood monocytes and neutrophils to circulate in the bloodstream [9,10], and bind to susceptible cells via a unique toxin-binding glycosphingolipid receptor [11]. Microvascular endothelial cells within the kidneys and central nervous system appear to be preferentially sensitive to the cytotoxic action of Shiga toxins. Once internalized, the toxins undergo retrograde intracellular trafficking to reach the lumen of the endoplasmic reticulum (ER) [12]. 

There are no vaccines to prevent disease caused by Stx-producing bacteria. Furthermore, treatment of the disease is primarily supportive, e.g., hemodialysis or peritoneal dialysis for HUS. Treatment with anti-motility agents and antibiotics are contraindicated in disease caused by Shiga toxins [1,2,3]. In order to define new targets for intervention in disease progression, extensive efforts have been undertaken by researchers to better understand the mechanism of action, intracellular trafficking, activation of host intracellular signaling pathways, and the elicitation of the innate immune response by Shiga toxins (reviewed in [13]). Interventional strategies currently under development to block disease caused by Shiga toxins may be categorized as: (i) toxin receptor analogues; (ii) toxin intracellular transport inhibitors; (iii) anti-toxin antibodies; and (iv) probiotic bacteria. Initial attempts to create a toxin receptor analogue involved the coupling of the Galα(1→4)Galβ(1→4)Glu trisaccharide of Gb3 to inert diatomaceous earth for oral administration to patients with bloody diarrhea [14]. This product is called Synsorb-Pk. The hypothesis was that Synsorb-Pk would bind free Shiga toxins in the intestinal tract and facilitate toxin removal. Unfortunately, Synsorb-Pk was not proven effective in clinical trials [15,16]. It was subsequently shown that Shiga toxins are capable of translocation across polarized intestinal epithelial monolayers [5,6] suggesting that toxin receptor analogues may be more efficacious if delivered intravenously. In this regard, Nishikawa and colleagues used carbosilane dendrimers to create highly branched compounds containing multiple Gb3 trisaccharides [17]. These compounds protected mice from a lethal dose of purified Stx2. Saenz *et al*. [18] screened over 14,000 low molecular compounds to identify two compounds that blocked Shiga toxin intracellular transport at different steps. One compound blocked toxin transport at the early endosome step, while the second compound blocked translocation through the Golgi apparatus. Several investigators have developed anti-Shiga toxin humanized monoclonal antibodies. Mukherjee *et al.* [19] screened a panel of ten anti-Stx1 humanized monoclonals and showed them all to be effective in neutralization of Stx1 *in vitro* and in protection of toxin challenged mice. A recombinant *E. coli* K-12 strain was engineered to express a modified LPS structure which binds Shiga toxins with high affinity [20]. When fed to mice, this recombinant strain protected the animals from oral challenge with Stx2-producing *E. coli.* Readers are referred to a recent review for more information on interventional strategies [13]. Despite these advancements, however, infections with Shiga toxin-producing bacteria are still a significant cause of morbidity and mortality. The development of effective drugs or vaccines to prevent or treat diseases caused by Shiga toxins will require an increased understanding of the interaction of the toxins with multiple cell types.

## 2. Characterization of Shiga Toxin Intracellular Transport

### 2.1. Toxin Structure and Function

The Shiga toxins constitute a family of genetically and functionally related cytotoxins. *Shigella dysenteriae* serotype 1 expresses the prototypical member of the family, Shiga toxin, and is the causative agent of bacillary dysentery. STEC, of which *Escherichia coli* O157:H7 strains are most common in many countries, express one or more toxins that are closely related to Shiga toxin [21]. However, non-O157 serotype enterohemorrhagic *Escherichia coli* (EHEC) have been associated with HUS and recently, a reference collection of HUS-associated EHEC (HUSEC) strains has been established by performing multilocus sequencing typing (MLST) [22]. Toxins expressed by STEC are antigenically categorized as Shiga toxin type 1 (Stx1), which is essentially identical to Shiga toxin, and Shiga toxin type 2 (Stx2), which possesses approximately 56% identity to Shiga toxin/Stx1 at the deduced amino acid sequence level [23]. A number of genetic variants of Stx1 and Stx2 have been characterized. X-ray crystallographic analyses revealed that all Shiga toxins contain six protein subunits in an AB_5_ molecular configuration. Thus, all Shiga toxins contain a single, enzymatic A-subunit in non-covalent association with a homopentamer of B-subunit proteins [24,25,26]. Toxin A-subunits are potent protein synthesis inhibitors which act by cleaving a single adenine residue from the 28S rRNA component of eukaryotic ribosomes [27,28]. The B-subunit proteins in the homopentamers of most of the holotoxins are able to bind to the neutral glycosphingolipid globotriaosylceramide (Gb3; also known as Gb3Cer, CD77 or the P^k^ blood group antigen) on the surface of host cells [11,29,30]. The only exception is the receptor for a genetic variant of Stx2, called Stx2e or pig edema disease toxin, which binds preferentially to the glycosphingolipid globotetraosylceramide (Gb4) [31].

### 2.2. Toxin Interaction with Cell Surface Receptors

The trisaccharide carbohydrate structure of Gb3 has been shown to directly interact with Shiga toxin B-subunits, although variability in the lipid moiety of Gb3 also affects toxin binding [32]. Interestingly, each B-subunit protein possesses three Gb3 binding sites, suggesting that each holotoxin molecule may cross-link up to 15 Gb3 molecules [33,34]. This cooperative binding may explain the high affinity of Shiga toxins for Gb3. Furthermore, Gb3 cross-linking may be a critical component for toxin mediated induction of negative membrane curvature and the initiation of toxin internalization [35]. Several lines of evidence established Gb3 as the receptor for Shiga toxins. The addition of Gb3 to culture medium inhibited the biological activity of Shiga toxins. In addition, when Gb3 receptors were destroyed by digestion of membrane glycosphingolipids with galactosidases, or if the synthesis of glycosphingolipids was blocked, toxin-mediated cytotoxicity was lost [36,37]. It is now recognized that Gb3 displays significant molecular heterogeneity, including differences in fatty acyl chain length, degree of bond saturation and hydroxylation status. The affinity of Shiga toxins for Gb3 is dependent on the structure of the fatty acid moiety, and hydroxylation of C22 and C18 fatty acids increased toxin binding to Gb3 [38]. Following Gb3 binding and the formation of toxin-containing membrane invaginations, the invaginations may form tubules that separate from the plasma membrane in a process called scission. Using giant unilamellar vesicles reconstituted with Gb3, Römer *et al*. [39] showed that α-hydroxylated Gb3 was necessary for scission. Vesicles containing non-hydroxylated Gb3 formed tubules which did not undergo scission. Finally, the nature of the phospholipids found in the surrounding membrane may also play a role in the interaction of Shiga toxins with Gb3 [40,41].

While the glycosphingolipid Gb3 is the major receptor responsible for toxin binding, leading to internalization and routing to the ER, Shiga toxins may associate with cells that lack membrane Gb3 expression. For example, Shiga toxins associate with human intestinal epithelial cells in such a manner as to allow the translocation of the toxins into the lamina propria, even though the human colonic epithelial lining is essentially Gb3 negative [42]. Shiga toxins have also been reported to bind an, as yet, uncharacterized receptor on Gb3-deficient human neutrophils, albeit with a lower affinity than the interaction with Gb3 [9,10,43]. Shiga toxin interaction with neutrophils does not appear to trigger toxin internalization or cytotoxicity. Numerous histopathological studies in HUS cases [44,45] and in animals receiving Shiga toxins [7,8], and *in vitro* studies [46,47], suggest that endothelial cells are the primary targets of the cytotoxic action of Shiga toxins. In general, cultured human endothelial cells express Gb3, bind Shiga toxins, and are susceptible to the cytotoxic effects of the toxins. However, endothelial cells derived from different tissues exhibit different susceptibilities to the toxins, and these differences in susceptibility may be linked with differences in Gb3 expression [48] and structural heterogeneity of Shiga toxin-binding glycosphingolipids [49]. Importantly, both from the standpoint of studies designed to explore toxin entry into cells and for studies on the progression of disease, expression of Gb3 on the cell surface may be regulated by many factors. Bacterial lipopolysaccharides (LPS), and the pro-inflammatory cytokines tumor necrosis factor-α (TNF-α) and interleukin 1 (IL-1) have all been reported to increase synthesis of Gb3 and exposure of toxin binding sites at the endothelial cell surface. In some cell types, membrane glycosphingolipid composition may be regulated by butyric acid, a metabolic end-product found in the digestive tract [11,50].

### 2.3. Intracellular Trafficking of the Toxins

Binding of the holotoxin molecule to Gb3 is a prerequisite for entry of the multi-subunit protein into the target cell [11]. In brief, once Shiga toxins bind Gb3, the toxins are internalized via the Golgi apparatus to the ER, a process referred to as retrograde transport [12]. The molecular mechanisms of intracellular transport of Shiga toxins have been reviewed in detail [30,51]. Also the use of the toxins as probes for studying intracellular transport and the B-subunit as a transporting vehicle for foreign proteins into target cells is reviewed by Sandvig *et al.* [51]. During intracellular trafficking, the A-subunits of Shiga toxins are cleaved by furin, a calcium-sensitive serine protease located in the *trans-*Golgi network [52,53]. The furin-processed A-subunit, comprised of a 27 kDa A1-fragment and a 4 kDa A2-fragment, remains linked via a disulfide bond. Within the ER, the A1- and A2-fragments separate [53]. The A1-fragment, containing *N*-glycosidase activity, is translocated across the ER membrane to cytoplasm. 

#### 2.3.1. Endocytosis of Shiga toxins

After binding to Gb3, Shiga toxins may be internalized by a clathrin-independent receptor-mediated process or internalized from clathrin-coated pits [29,30,54]. The toxins have been shown capable of mediating clathrin phosphorylation, which triggers toxin uptake in some cell types [55]. The mechanism of clathrin-independent toxin internalization remains to be clarified, although Römer *et al.* [35] reported that B-subunits of Shiga toxin induce membrane invaginations with the development of tubular connections for toxin cellular uptake. Interestingly, it was recently reported that the role of clathrin for endocytosis and intracellular transport of the toxins may be altered in a single cell type. Specifically, butyric acid treatment of HeLa and BHK cells not only sensitized the cells to the cytotoxic action of Shiga toxins, but also increased internalization via clathrin-dependent mechanisms [56]. Confocal microscopy experiments revealed a higher extent of co-localization between Shiga toxins and clathrin after butyric acid treatment. In HeLa cells, Shiga toxin B-subunits were found to be associated with detergent-resistant membrane microdomains or lipid rafts [57]. To what extent such an association is important for the endocytic pathway utilized by the toxins is not known, although macrophages, which do not coalesce Gb3 into lipid rafts, fail to route toxin B-subunits to the ER [57]. Thus, after endocytosis, Shiga toxins or toxin B-subunits may be: (i) directly transported from early/recycled endosomes to the Golgi apparatus for further transport to the ER [58,59]; (ii) transported to lysosomes for proteolytic degradation [57]; or (iii) transcytosed across polarized epithelial monolayers [5,6]. 

#### 2.3.2. Endosome to Golgi Apparatus Transport of Shiga Toxins

A critical step in the intracellular transport of Shiga toxins leading to intoxication and cell death is the transport of the toxins from early endosomes to the *trans*-Golgi network (TGN). A well-studied pathway leading from early endosomes to the Golgi apparatus involves transport via the late endosome utilizing the small GTP-binding protein Rab9. This Rab9-dependent pathway is responsible for sorting mannose-6-phosphate receptors (M6PR) [60,61,62]. This pathway is also used by furin, a protease which processes Shiga toxin A-subunits, that recycles between the cell surface, endosomes, and the Golgi apparatus [63]. In contrast to this well characterized transport process, Shiga toxins appear to utilize a Rab9-independent pathway for efficient transport from endosomes to the TGN using a mechanism dependent on functional dynamin and clathrin [12,56]. In particular, clathrin adaptor protein epsinR is involved in sorting from early endosomes to the *trans*-Golgi network (TGN) in the retrograde transport of Shiga toxins [64]. Additional host proteins involved in toxin transport to the Golgi apparatus include: (i) Rab11 and a Rab6 isoform (Rab6a’), GTP-binding proteins which also regulate vesicular trafficking; (ii) v-soluble N-ethylmaleimide-sensitive factor attachment protein receptor (v-SNARE), described as a trans-membrane protein essential for membrane fusion [65]; and (iii) vesicle-associated membrane protein (VAMP) 3, and VAMP4 [66,67,68]. The requirement of the retromer complex in retrograde transport was established by using the B-subunit of Shiga toxin [69]. Recently, Bujny *et al*. [70] and Utskarpen *et al*. [71] defined an essential role of protein retromer component sorting nexin-1 (SNX1) and sorting nexin-2 (SNX2) for efficient trafficking of Shiga toxin from early endosomes to the TGN. A complete understanding of the sorting mechanisms trafficking Shiga toxins from early endosomes to the Golgi apparatus is still an area of active investigation. 

#### 2.3.3. Toxin Transport from the *Trans*-Golgi Network to the ER and Nuclear Envelope

Studies are ongoing to describe the mechanisms by which Shiga toxins are transported in a retrograde manner from the TGN to the ER. Normally, host proteins may be retrieved from the Golgi to the ER, and retrotranslocated in the ER, if the proteins possess a KDEL sequence capable of binding so-called KDEL receptors in the Golgi apparatus. For bacterial toxins, such as *Pseudomonas* exotoxin A, which possess KDEL or KDEL-like sequences, transport via interaction with the KDEL-receptor in COP I-coated vesicles is involved in retrograde transport to the ER [72]. Despite the fact that Shiga toxins do not express the KDEL retrieval sequence motif, they are able to move in a retrograde manner from the Golgi apparatus to the ER via a COP I-independent, Rab6a’-dependent route [73,74]. Shiga toxin 1 has been shown to reach the nuclear envelope [41,75] and to get into the nucleus of intoxicated cells [76] inducing the formation of apurinic/apyrimidinic sites in nuclear DNA [77].

#### 2.3.4. Transport of Shiga Toxins from the ER to the Cytosol

The characterization of translocation mechanisms by which Shiga toxins move from the ER lumen into the cytosol is an area of active study. Internalization of many AB toxins, such as large clostridial cytotoxins, results in the formation of a channel in the endosomal membrane so that toxin enzymatic A-subunits may be directly delivered into the cytosol for host cell intoxication [78]. However, several AB_5_ toxins, such as Shiga toxins and cholera toxin, cannot form pores for the purpose of delivery of toxin A-subunits into the cytosol [79,80]. Within the ER, the Shiga toxin A1-fragment dissociates from the A2-fragment + B-subunits following proteolysis by furin and disulfide bond reduction [30,53]. Toxin mutants with deletions in the furin cleavage site are processed by a calpain-like enzyme [52,81], suggesting that proteases other than furin may process Shiga toxin A-subunits for retrotranslocation across the ER membrane. However, alternative mechanisms of A-subunit processing remain to be rigorously defined. Finally, several studies have shown that Shiga toxins appear to interact with ER lumen-resident chaperones such as HEDJ/ERdj3 [82] and BiP [83]. Interactions with chaperones may be necessary to induce unfolding of the A1-fragment prior to retrotranslocation across the ER membrane. To date, published studies suggest that only the toxin A1-fragment is retrotranslocated into the cytoplasm [84]. 

## 3. Shiga Toxins Induce the Ribotoxic Stress Response

Despite advances in understanding toxin structure, mode of action, and molecular mechanisms of toxin intracellular transport that underlie the pathogenesis of diseases caused by Shiga toxins, investigation of the action of the toxins on the 60S component of eukaryotic ribosomes brought attention to novel properties of the toxins which may or may not contribute to protein synthesis inhibition and cytotoxicity. In 1997, Iordanov *et al*. [85] reported that two toxic enzymes which act on eukaryotic ribosomes, the plant toxic lectin ricin A-chain and the fungal ribotoxin α-sarcin, targeted the peptidyl transferase reaction center of 28S rRNA for inactivation of ribosomes and protein synthesis inhibition, and in the process, activated the stress-activated protein kinase signaling pathway involving c-Jun NH_2_-terminal kinases (JNK). Shiga toxin A1-fragments, through their *N-*glycosidase activity, remove a single adenine residue from a portion of the 28S rRNA molecule involved in the peptidyl transferase reaction. It was subsequently shown using human epithelial and monocytic cell lines [86,87,88] that Shiga toxin-mediated modification of the ribosome triggered not only the activation of the JNK pathway, but the activation of the p38 mitogen-activated protein kinase (p38 MAPK) and extracellular-signaling regulated kinase (ERK) pathways. MAPK activation originating from toxin-modified ribosomes has been termed the ribotoxic stress response [85]. Shiga toxin-mediated activation of the ribotoxic stress response in epithelial and macrophage-like cells appears to be essential for the expression of pro-inflammatory cytokines and chemokines [86,87,88]. Double-stranded RNA-activated protein kinase R (PKR), and the upstream kinases MKK 3/6 and ZAK, participate in signaling during the ribotoxic stress response induced by ricin, Shiga toxins, and the fungal trichothecene toxin deoxynivalenol (DON) [89,90]. The role of ribotoxic stress response in induction of apoptosis by Shiga toxins remains to be fully characterized. Smith *et al*. [87] showed that treatment of epithelial cells with a JNK or p38 MAPK inhibitor protected cells from cytotoxicity and reduced procaspase-3 activation. JNK, p38 and ERK MAPKs also appear to regulate the differential phosphorylation of the anti-apoptotic factor Bcl-2 [91,92] so that apoptosis may be facilitated or inhibited. Finally, protein synthesis *per se* does not induce the ribotoxic stress response. For example, cycloheximide, a drug which effectively inhibits protein synthesis in human monocytes, does not induce the ribotoxic stress response or induce apoptosis [93]. Thus, the mechanism by which protein synthesis is inhibited, *i.e.*, acting on the peptidyl transferase center, may be essential for ribotoxic stress response activation. 

## 4. ER Stress Responses and Shiga Toxin-Induced Apoptosis

### 4.1. Shiga Toxins Induce Apoptosis

Apoptosis, or programmed cell death, is a type of cell death originally described by morphological changes such as membrane blebbing (formation of cellular apoptotic bodies), cell shrinkage, and nuclear changes including chromatin condensation and DNA fragmentation. Apoptosis is mediated by sequential activation of a cascade of aspartate-specific cysteine proteases, called caspases. Two major pathways involved in the induction of apoptosis have been characterized: the intrinsic or mitochondria-mediated, and the extrinsic or death receptor-mediated pathways of apoptosis induction. Recently, numerous studies have shown that Shiga toxins induce apoptosis through different mechanisms in different cell types, including human epithelial cells, endothelial cells, and neurons (reviewed in [94]). Primary human monocyte-derived macrophages appear to be resistant to intoxication by Shiga toxins at physiological concentrations when cultured *in vitro* [95]. However, Stx1 induces apoptosis of the human myeloid leukemia cell line THP-1 in a cell maturation-dependent manner [96]. Stx1-mediated apoptosis of monocytic THP-1 cells involved a novel pathway using components of both the extrinsic and intrinsic pathways of apoptosis [93]. Stx1 induced the cleavage of procaspase-8 to its active form caspase-8, which appeared to be an early event, leading to the direct activation of procaspase-3 and the generation of the activated form of the Bcl-2 family protein BID (truncated BID or tBID). tBID translocated to the mitochondrial membrane leading to increased mitochondrial membrane permeability, release of cytochrome *c* and formation of the apoptosome. Stx1 enzymatic activity was required to induce apoptosis. A significant fraction of procaspase-8 is known to be associated with the ER membrane [97] and this ER-localized procaspase-8 may be involved in the early activation of caspase-8. Taken together, the data indicated that apoptosis of monocytic THP-1 cells required the retrograde transport of functional toxin to the ER, where signaling for cell death through early procaspase-8 cleavage may originate. 

### 4.2. Shiga Toxins Induce the ER Stress Response Leading to Apoptosis

In addition to its role in the translocation of nascent proteins into the cell secretory apparatus, the ER is the principal site for the correct folding of proteins. A protein “quality control” process which monitors protein folding, and initiates a highly orchestrated series of signaling events to properly fold proteins, has been characterized [98,99]. Activation of this “quality control” process includes ER luminal proteins called molecular chaperones. Chaperones associate with improperly folded proteins and may facilitate protein re-folding or retrotranslocation of mis-folded proteins into the ER-associated degradation (ERAD) pathway [100,101]. The rate of flux of newly synthesized proteins through the ER may vary based upon environmental signals, such as alterations in the ER lumen redox state, aberrant glycosylation, aberrant intracellular calcium homeostasis, and temperature. Thus, the capacity of the ER to correctly fold nascent proteins may become saturated, and this condition is referred to as ER stress [102,103]. The three proximal ER membrane-associated sensors of protein folding are the kinase PKR-like ER kinase (PERK), the kinase/endoribonuclease inositol-requiring enzyme 1 (IRE-1) and the transcription factor activating transcription factor-6 (ATF-6) [103]. Normally, these sensors are thought to be in association with the chaperone BiP. However, in the presence of improperly folded proteins, BiP dissociates from PERK, IRE-1 and ATF-6. PERK and IRE-1 are thought to undergo proximity-dependent auto-phosphorylation, while ATF-6 translocates to the Golgi apparatus and undergoes proteolytic processing to form an active transcription factor [103,104,105,106]. PERK-mediated phosphorylation of the translation initiation factor eIF2α transiently inhibits most protein translation, thereby reducing the flux of nascent polypeptides through the stressed ER [104]. However, ER stress activates genes possessing promoters with ER-stress response elements (ERSE) and unfolded protein response elements (UPRE) [106]. The transcriptional activation through these promoter elements modulates the expression levels of ER-resident chaperones, such as BiP, involved in assisting correct protein folding in the ER [107]. Increased transcriptional activation is mediated, in large part, by IRE-1 and ATF-6. The endoribonuclease function of IRE-1 cleaves the transcript for the transcription factor X-box protein-1 (Xbp-1), and the processed mRNA encodes a transcription factor with an altered DNA binding profile [108,109]. ATF-6 is cleaved by host cell proteases S1P and S2P to form an active basic leucine zipper transcription factor [110]. Thus, even in the face of translational inhibition, a subset of genes encoding proteins involved in protein folding and processing are expressed. 

**Figure 1:**
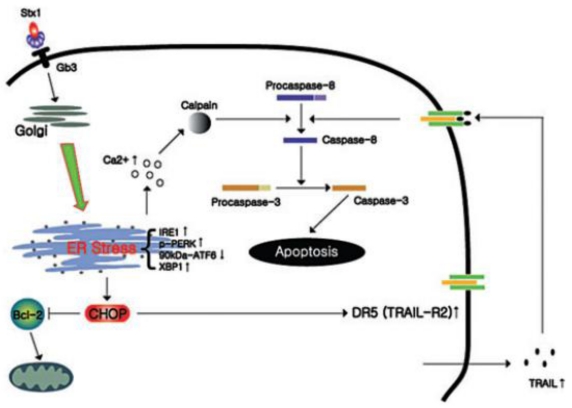
Model of Stx1-mediated ER stress and induction of apoptosis. Following binding to the membrane glycosphingolipid receptor Gb3, Stx1 is transported to the ER. The ER stress sensors IRE1, PERK are activated by phosphorylation (up arrows) while levels of unprocessed 90 kDa ATF-6 decrease (down arrow) leading to release of the active 50 kDa active transcription factor. Signaling through the ER stress sensors leads to increased CHOP, DR5 and TRAIL expression, and decreased Bcl-2 expression. Autocrine or paracrine TRAIL–DR5 interactions may contribute to apoptosis induction. Toxin transport to the ER is also associated with increased cytosolic Ca^2+^ levels, leading to calpain activation and cleavage of procaspase-8 to produce the initiator caspase and activation of programmed cell death. Reproduced with permission from Wiley [113].

The ER stress response has been reported in be initiated in pathogen-infected cells [111,112], and given the capacity of Shiga toxins to inhibit protein synthesis and generate truncated or mis-folded proteins, one might predict that the toxins would activate the ER stress response. Lee *et al*. [113] demonstrated that Stx1 treatment of the human monocytic cell line THP-1 was capable of inducing ER stress and activating all three proximal ER stress sensor molecules involved in the immediate detection of improperly folded proteins (Figure 1). Furthermore, all the sensors were active as shown by phosphorylation of eIF2α, processing of Xbp-1 mRNA, and the detection of ATF-6 cleavage fragments. 

Multiple outcomes may follow activation of the ER stress response. If the accumulation of improperly folded proteins is cleared from the ER, the cell may return to normal homeostasis. If activation of the ER stress response fails to clear the cause of stress, apoptotic cell death may follow (reviewed in [107]). Thus, prolonged signaling through PERK, IRE-1 and ATF-6 may activate programmed cell death. PERK- and ATF-6-dependent up-regulation of the gene encoding C/EBP homology protein (CHOP; alternatively referred to as GADD153) appears essential in apoptosis induction. CHOP is a transcriptional regulator that represses the expression of anti-apoptotic factor Bcl-2 [107,114]. Stx1 treatment of toxin sensitive monocytic THP-1 cells led to increased CHOP expression and decreased Bcl-2 expression (Figure 1) [113]. In agreement with earlier studies characterizing signaling mechanisms activated by ER stress leading to apoptosis [115,116], Stx1-induced up-regulation of CHOP also increased the expression of the apoptosis ligand-death receptor pair TNF-α-related apoptosis inducing ligand (TRAIL) and death-domain containing receptor 5 (DR5, also referred to as TRAIL-R2) [113]. Thus, monocytic THP-1 cells intoxicated with Shiga toxins appear incapable of alleviating ER stress leading to apoptosis.

Shiga toxins are not the only microbial toxins that may induce apoptosis through prolonged activation of the ER stress response. It has recently been shown that subtilase cytotoxin, a new member of the AB_5_ toxin family which is secreted by some STEC, cleaves the chaperone BiP, thereby triggering the ER stress response resulting in cytotoxicity [117,118]. Subtilase cytotoxin activates all the proximal sensors of ER stress [118]. Deoxynivalenol, a trichothecene mycotoxin produced by *Fusarium* sp., also triggers the ER stress response via IRE1, XBP-1 and ATF-6 activation and causes degradation of BiP in murine peritoneal macrophages [119].

### 4.3. Calcium Involvement in Shiga Toxin-Induced Apoptosis

The role of calcium (Ca^2+^) influx from extracellular sources or efflux from intracellular stores in the decision to commit to apoptosis or cell survival in response to extracellular stress has been explored for more than two decades [120,121]. In response to ER stress, Ca^2+^ in intracellular stores may be released into the cytoplasm from the ER. Bax and Bak, Bcl-2 protein family members containing BH3 domains, are ER membrane localized and participate in rapid Ca^2+^ efflux. Ca^2+^ may then be taken up by mitochondria or may directly activate the calcium-dependent proteases called calpains. The translocation of Bax and Bak to mitochondrial membranes may lead to increased membrane permeability and generation of the apoptosome, and calpains may directly activate caspases. Thus, changes in intracellular Ca^2+^ localization may contribute to the induction of apoptosis [122,123,124]. Stx1 perturbs normal intracellular Ca^2+^ homeostasis by triggering Ca^2+^ release from ER stores into the cytoplasm in THP-1 cells [113,125]. Cherla *et al*. [125] showed that Stx1 activated PI3K, which may trigger generation of phospholipid second messengers and release of Ca^2+^ from intracellular stores in macrophage-like THP-1 cells. However, our understanding of the role of intracellular Ca^2+^ flux and Ca^2+^-activated regulatory components in apoptosis induction in Shiga toxin-stressed cells will require further studies. 

## 5. Shiga Toxin-Induced ER Stress: The Cell Death *vs.* Survival Decision

Relatively little is known about the regulatory mechanisms by which host cells alter signaling pathways in response to Shiga toxins to induce apoptosis or favor cell survival. As noted earlier, sensitivity of the THP-1 cell line to the cytotoxic action of Shiga toxins is cell maturation-dependent [96]. Undifferentiated, monocytic THP-1 cells are sensitive to killing by purified Stx1, while differentiated, macrophage-like THP-1 cells are relatively refractory to the cytotoxic action of the toxin, with only approximately 30% of cells undergoing apoptosis. Interestingly, apoptotic signaling pathways were activated by Shiga toxins in both cell types, suggesting that compensatory cell survival signaling pathways may be triggered in mature, macrophage-like cells, but not in monocytic cells [126,127]. In studies designed to explore apoptotic and cell survival signaling pathways triggered by Stx1 in THP-1 cells, differences in activation of the ER stress response were noted. Specifically, Stx1 activated the proximal ER stress sensors PERK and IRE-1, but failed to mediate ATF-6 proteolysis in macrophage-like cells [127]. Thus, one of the main regulators of CHOP expression was not activated in Stx1 treated macrophage-like THP-1 cells. Pro-apoptotic signaling pathways, including increased TRAIL and DR5 expression, and release of Ca^2+^ from intracellular stores and calpain activation, which were initially characterized in the toxin sensitive monocytic cell, were also activated in toxin resistant macrophage-like THP-1 cells. However, compensatory pro-survival signaling mechanisms appeared to be selectively activated in macrophage-like cells. In specific, expression levels of the pro-survival factor Bcl-2 were increased, Bcl-2 readily translocated to the mitochondria, and Bcl-2 was phosphorylated at serine residues which facilitate pro-survival functions of Bcl-2 [127]. Thus, as has been reported earlier [128,129], the balance between cell death and survival signaling appears to be regulated by the interplay with pro-apoptotic factors, such as Bax/Bak, with pro-survival factors such as Bcl-2 and Bcl-xL. To explore the mechanisms of apoptosis induction in the subset of macrophage-like THP-1 cells that die in response to Stx1, we utilized RNA interference technology to knock-down DR5 and CHOP expression levels. The data indicated that silencing these pathways significantly protected cells against Stx1 treatment in differentiated macrophage-like cells, but not monocytic cells, suggesting that the TRAIL-DR5 pathway may be relatively more important in killing differentiated cells [130]. We also noted differences in the kinetics of calpain activation in Stx1-treated undifferentiated and differentiated cells. Calpain activation occurred rapidly after intoxication in undifferentiated monocytic cells, suggesting that Ca^2+^-dependent signaling may account for the heightened toxin sensitivity.

## 6. Conclusions

Structures of Shiga toxins, toxin enzymatic activity, and toxin intracellular trafficking have been defined. However, only limited studies have been published on the host cellular responses triggered or regulated during Shiga toxin binding, internalization and trafficking. Following interaction with the toxin-binding glycosphingolipid Gb3, Shiga toxins are internalized, trafficked into and through the Golgi apparatus in a retrograde manner to reach the lumen of the ER. From the ER, a proteolytically processed fragment of the toxin A-subunit enters the host cell cytosol and catalytically cleaves single adenine residues from the 28S rRNA component of ribosomes. This enzymatic activity, in addition to mediating host cellular protein synthesis inhibition, also activates the ribotoxic stress response and the ER stress response. The capacity of Shiga toxins to induce prolonged signaling through the ER stress response may lead to apoptosis. Apoptotic pathways activated by Shiga toxins include components of the intrinsic (mitochondria-mediated) and extrinsic (DR5-TRAIL system) pathways. However, Shiga toxins may also activate cell survival pathways, primarily through mechanisms involving the pro-survival Bcl-2 proteins. Taken together, perturbing the intracellular homeostasis between pro-apoptotic and pro-survival signaling molecules represents a critical determinant in the cell death/cell survival decision following intoxication with Shiga toxins. An improved understanding of host cell responses to the toxins will be necessary to develop therapeutic strategies to intervene in disease progression caused by the toxins. 
